# 

**Published:** 1893-01

**Authors:** 


CONTENTS.

VOL. 40. No. 1.

| | PAGE. |
|---|---|
| The New Year | 1 |
| When to Eat | 3 |
| Perils of Investigation | 5 |
| Behind the Counter | 7 |
| Rest and Disease | 8 |
| The Opium Curse | 9 |
| Physical Perfection | 11 |
| Rapid Motion of the Blood | 11 |
| Gray Hairs | 12 |
| Infectiousness of Tuberculosis | 13 |
| The Eyes | 14 |
| Care of the Hands | 14 |
| A Crimean Ghost Story | 15 |
| Germ Diseases | 17 |
| Death by Violence | 17 |
| Prevention of Juvenile Smoking | 18 |
| Sleep | 19 |
| The Sultan's Food | 19 |
| Miscellaneous | 20 |
| Literary | 22 |
| A Fragment | 24 |

INDEX TO ADVERTISEMENTS OF HALF A PAGE AND OVER.

| | PAGE. |
|---|---|
| Dr. Jaeger's Sanitary Woolens | I |
| The World Almanac | II |
| The Press Claims Co. | III |
| The Rip Van Winkle Rocker | IV |
| James Vick's Sons | V |
| Hall's Journal Premiums. | VII |
| Washington Improvement Co. | VIII |
| J. Fehr's Compound Talcum. | IX |
| Herba Vita | X |
| Royal Baking Powder | XI |
| Buffalo Lithia Water | XI |

